# Detection of β-methylphenethylamine, a novel doping substance, by means of UPLC/MS/MS

**DOI:** 10.1007/s00216-014-7728-5

**Published:** 2014-03-16

**Authors:** Piotr Chołbiński, Mariola Wicka, Katarzyna Kowalczyk, Anna Jarek, Paweł Kaliszewski, Andrzej Pokrywka, Ewa Bulska, Dorota Kwiatkowska

**Affiliations:** 1 Department of Anti-Doping Research, Institute of Sport, Trylogii 2/16, 01-982 Warsaw, Poland; 2 Faculty of Chemistry, Biological and Chemical Research Center, University of Warsaw, Żwirki i Wigury 101, 02-083 Warsaw, Poland

**Keywords:** Stimulant, Amphetamine isomer, Liquid chromatography, Mass spectrometry, Doping, Sport

## Abstract

Novel substances of expected doping activity are constantly introduced to the market. β-Methylphenethylamine (BMPEA) is classified as a doping agent by the World Anti-Doping Agency as it is a positional isomer of amphetamine. In this work, the development and application of a simple and rapid analytical procedure that enables discrimination between both isomers is described. The analytes of interest were extracted from urine by a two-step liquid–liquid extraction and then analyzed by UPLC/MS/MS under isocratic conditions. The entire analytical procedure was validated by evaluating its selectivity, discrimination capabilities, carry-over, sensitivity, and influence of matrix effects on its performance. Application of the method resulted in detection of BMPEA in eight anti-doping samples, including the first report of adverse analytical finding regarding its use. Further analysis showed that BMPEA may be eliminated unchanged along with its phase II conjugates, the hydrolysis of which may considerably improve detection capabilities of the method. Omission of the hydrolysis step may therefore, produce false-negative results. Testing laboratories should also carefully examine their LC/MS/MS-based amphetamine and BMPEA findings as both isomers fragment yielding comparable collision-induced dissociation spectra and their insufficient chromatographic separation may result in misidentification. This is of great importance in case of forensic analyses as BMPEA is not controlled by the public law, and its manufacturing, distribution, and use are legal.

## Introduction

In recent years, new doping substances have been" continuously introduced to the market in the form of nutritional supplements. They are often produced in clandestine drug laboratories by modification or positional rearrangement of well-established doping agents such as stimulants [1–3]. The main aim of these activities is to deliver specifically designed, biologically active substances which are not controlled by the public law. This allows for making a profit on selling a product yielding “unprecedented results” that, in the case of stimulating agents, would correspond to such effects as rapid weight loss or the ability to perform extensive training for extended periods of time. The presence of designer substances in these products usually remains unknown until anti-doping laboratories identify them [1, 4–6]. This, in turn, may lead to a large number of sanctioned athletes that fail anti-doping tests once testing laboratories have implemented methods for their detection. This is due to the fact that the list of substances prohibited in sport is open and defines novel doping agents based on their similarity in action and/or structure to those already listed [7, 8]. One of the recent examples is 1,3-dimethylamylamine [9, 10], an isomer of the stimulant tuaminoheptane.

Athletes invariably claim that they were unaware of the presence of new designer stimulants in the supplements they have used. In fact, these agents are often not listed in the nutrition fact labels, or even if they are, their chemical names are either altered or are too difficult for a non-chemist to identify them. In the case of 1,3-dimethylamylamine, the labels listed either its synonyms (37 deposited in the PubChem database [11]) or named geranium oil or geranium extract as its source. Although a notion that this substance was added to nutritional supplements in a form of synthetic material seems to predominate, there is still an intense debate whether or not these extracts indeed contain 1,3-dimethylamylamine [12–15]. The mechanism of action of newly designed substances is usually unknown; they are simply expected to have a similar biological effect to the template substance of desired activity [2, 3]. As such, they may pose a serious threat to public health, and e.g., death cases having possible associations with 1,3-dimethylamylamine use have already been reported [16]. Forensic analyses performed in relation to such fatal accidents must unequivocally identify the substance that may have been abused or ingested by the deceased. This, in turn, allows for legal actions to be taken against the manufacturers or distributors. However, this is mostly the case when these substances are listed as controlled by the government. It is also noteworthy that returning an adverse finding related to inadvertent doping may have detrimental effects on the mental balance of professional athletes, especially those that are rather risk avoiders and value their health over the medal winning [17].

β-methylphenethylamine (BMPEA) is a novel additive of expected stimulant activity found in nutritional supplements. It is a positional isomer of amphetamine, and based on this structural similarity, it is classified as a stimulant by the World Anti-Doping Agency (WADA) [7]. Detection of BMPEA or amphetamine in a urine sample collected in competition constitutes a doping offense and results in sanctions against the athlete. In the forensic field, however, unequivocal identification of amphetamine in a mixture of its positional isomers and structurally related compounds is of critical importance; the latter substances are often not controlled by the public law, and their manufacturing, distribution, and use are legal.

The aim of this work was to elaborate an ultra-performance liquid chromatography–tandem mass spectrometric (UPLC/MS/MS) analytical procedure enabling discrimination between amphetamine and BMPEA. The described method was successfully applied to anti-doping urine samples and led to the report of the first adverse analytical finding regarding BMPEA use.

## Experimental

### Chemical and reagents

The standards of pure substances amphetamine, β-methylphenethylamine (1-amino-2-phenylpropane), and phentermine were purchased from Sigma-Aldrich (Poland). Hydrochloric acid, potassium hydroxide, sodium sulfate, and boric acid were obtained from POCH (Poland). l-Cysteine, *tert*-butanol, and LC/MS-grade methanol were purchased from Merck Millipore (Germany). Diethyl ether and formic acid were from J.T.Baker (Holland), whereas methyl *tert*-butyl ether (MTBE) was purchased from Rathburn (Scotland). The Millipore DirectQ UV3 system (*R* > 18 MΩ/cm, Germany) was used as the source of water.

Stock solutions of standard substances were prepared at the concentration of 1 mg/ml in methanol and stored at −20 °C. Working solutions were prepared in methanol at the concentrations of 10 ng/ml, 1 μg/ml, or 100 μg/ml and were stored at 4 °C.

### Sample preparation

Samples were prepared according to the procedure described previously [18] with modifications. Briefly, 1 ml of urine was diluted with 4 ml of water and subsequently spiked with 50 μl of phentermine at 1 μg/ml (internal standard). After an addition of 1 ml of 6 mol/l HCl and approx. 100 mg of l-cysteine (antioxidant), samples were incubated at 105 °C for 30 min and cooled down to room temperature afterwards. Next, extraction with 5 ml of diethyl ether was performed (20 min) in order to remove acidic interferences. Samples were then centrifuged (5 min/3,000 rpm), and the ether phase was discarded. The pH of aqueous phase was adjusted to 9–10 with 1 ml of 10 mol/l borate buffer, and this was followed by the addition of 500 μl of *tert*-butanol, approx. 3 g of anhydrous sodium sulfate, and 5 ml of MTBE. Samples were then shaken and centrifuged, and the organic layer was recovered and evaporated under a nitrogen flow at 55 °C. The dry residue was reconstituted in 1 ml of methanol/H_2_O mixture (*v*/*v*, 2:8). Injection volume was fixed at 5 μl.

To assess whether conjugate hydrolysis improves the method performance, excretion samples were spiked with amphetamine at 300 ng/ml (internal standard) and prepared in duplicates according to the protocol described above with one exception: one sample of each duplicate was kept in a heat block for 30 min without the addition of acid in order to considerably shorten the duration of hydrolysis. Afterwards, the samples were cooled down to room temperature, and 1 ml of 6 mol/l HCl was added to reproduce the extraction conditions of the cleanup step.

### Chromatographic separation

Chromatographic separation was achieved on a Waters Acquity UPLC system equipped with an HSS T3 column (100 mm × 2.1 mm, 1.7 μm; Waters, USA). The mobile phase consisted of 0.1 % formic acid in water (A) and 0.1 % formic acid in methanol (B), and the flow rate was 300 μl/min at 45 °C. The initial B concentration of 10 % was constant over 7.5 min to resolve the isomers. Next, it increased to 100 % in 1 min and then was held for additional 0.5 min. The column was re-equilibrated with the mobile phase of the initial composition for 1.5 min. Samples were stored at 4 °C in an autosampler prior to analysis.

### Mass spectrometry conditions

The studied substances were traced in a multiple reaction monitoring (MRM) mode with a Micromass Quattro Premier XE (Waters, USA) mass spectrometer equipped in an electrospray ionization (ESI) source. The desolvation gas flow was set at 800 l/h and 300 °C, and the source temperature was 120 °C. The capillary voltage applied was 2.0 kV. The cone and collision gas flows were set at 50 l/h and 0.35 ml/min, respectively. Dwell time was set at 0.01 s. Amphetamine and BMPEA were traced at the cone voltage (CV) set at 20 V with the following selected precursor ion–product ion transitions at their respective collision energies (CE): *m*/*z* 136.11 > 65.04, CE 35 eV; 136.11 > 91.05, CE 20 eV; and 136.11 > 119.09, CE 10 eV. To monitor phentermine, the 150.13 > 91.05 transition was used at the CV and CE set at 25 V and 25 eV, respectively.

Collision-induced dissociation spectra were obtained with a Micromass QToF Premier mass spectrometer (Waters, USA) equipped in an ESI source. The desolvation gas flow was set at 600 l/h and 300 °C, and the source temperature was 120 °C. The cone and collision gas flows were set at 20 l/h and 0.15 ml/min, respectively. The system was operated in a wide-pass quadrupole mode, and data were acquired in a W-optics centroid mode over the range of *m*/*z* 30–300 with the mass resolution of at least 14,000 full width at half maximum (FWHM). The scan time was 0.5 s with the interscan delay set at 0.02 s. The capillary and cone voltages, and collision energies applied were set individually for every substance. Solutions of reference substances at 100 μg/ml in H_2_O/methanol (*v*/*v*, 1:1) were infused at the rate of 10 μl/ml in the continuous flow of the mobile phase (90 μl/ml) and then into the system.

### Method validation

#### Selectivity

Method selectivity was studied by analyzing 10 different urine specimens known to be free of amphetamine and BMPEA. The extracted ion chromatograms at the retention times of the studied compounds were examined for interfering peaks.

#### Discrimination capability and limit of detection

Identification criteria and minimum required performance limits (MRPLs) applied for the method followed the TD2010IDCR [19] and TD2013MRPL [20] technical documents, respectively. In order to evaluate the capability of the method to discriminate between amphetamine and BMPEA at different concentration levels, each of six blank urine samples was spiked with both isomers at 10 ng/ml (0.1 MRPL), 50 ng/ml (0.5 MRPL), 250 ng/ml (2.5 MRPL), and 1,250 ng/ml (12.5 MRPL). The extracted ion chromatograms were then inspected in order to assess whether obtained results allowed for unequivocal identification of the substances.

#### Recovery, matrix effect, and process efficiency

Investigation of recovery (RE), matrix effect (ME), and process efficiency (PE) was performed in six different urine matrices at two concentration levels and followed the procedure published by Matuszewski et al. [21] and updated by Marchi et al. [22]. To evaluate RE, urine samples were fortified with analytes at 50 ng/ml and 1,250 ng/ml and extracted together with two blank samples for each urine. The latter samples were then spiked with the analytes at the corresponding concentrations just prior to evaporation. The RE was calculated by the comparison of peak areas obtained for samples fortified before and after extraction. ME was determined by dividing peak areas recorded for samples spiked after extraction by peak areas of corresponding standard samples prepared in the mobile phase. Finally, to establish PE, peak areas obtained for samples fortified before extraction were compared to the peak areas of corresponding standard samples prepared in the mobile phase. All parameters were expressed as percentages.

#### Carry-over

Carry-over was evaluated by three consecutive injections of blank samples after a sample spiked post-extraction with analytes at 1,250 ng/ml. The analysis was performed for two different urine samples. The presence of carry-over was evaluated by visual inspection of the chromatograms obtained for blank urine samples. A ratio of peak areas of the blank sample and the sample containing analytes was defined as satisfactory when its value was below 0.1 %.

### Application to real case samples

Following the validation, the method was routinely used to confirm the presence of amphetamine and BMPEA in suspected anti-doping samples. Four samples found positive for BMPEA were subsequently used to evaluate the impact of glucuronide and sulfate deconjugation on the method performance. Samples were prepared in duplicates with the addition of amphetamine at 300 ng/ml (internal standard), and one sample for each duplicate was hydrolyzed for a shorter time. Concentrations of BMPEA were calculated by comparing the peak areas of BMPEA and amphetamine in a given sample.

### Dilute-and-shoot analysis

Samples were prepared as follows: 500 μl of urine was diluted with 500 μl of water and then spiked with 10 μl of phentermine at 10 μg/ml as the internal standard. Samples were briefly vortexed and centrifuged (5 min/3,000 rpm), and 100 μl was transferred to vials afterwards. Injection volume was fixed at 10 μl. Selectivity was determined by the analysis of 20 urine samples that had been deemed negative in the routine anti-doping screening. Limits of detection were established in 10 different urines, each spiked with amphetamine and BMPEA at 10 or 50 ng/ml. For analysis of excretion samples, amphetamine (final concentration, 300 ng/ml) was used as an internal standard both to quantify BMPEA and to correct for considerable variations in the BMPEA retention time caused by the matrix components. The analysis of excretion samples was repeated three times.

## Results and discussion

A growing market of designer stimulants leads to the detection of such new compounds by anti-doping laboratories almost every year. As these substances are often functional and/or structural analogs of already known doping agents, their use by an athlete in sport competitions constitutes a doping offense. In respect of the detection methodology, structural analogs often exhibit patterns of collision-induced dissociation (CID) and retention times comparable with those of well-established stimulants. This, in turn, gives anti-doping laboratories a possibility to identify them even in directed screening procedures.

### Development of confirmatory method

**Fig. 1:**
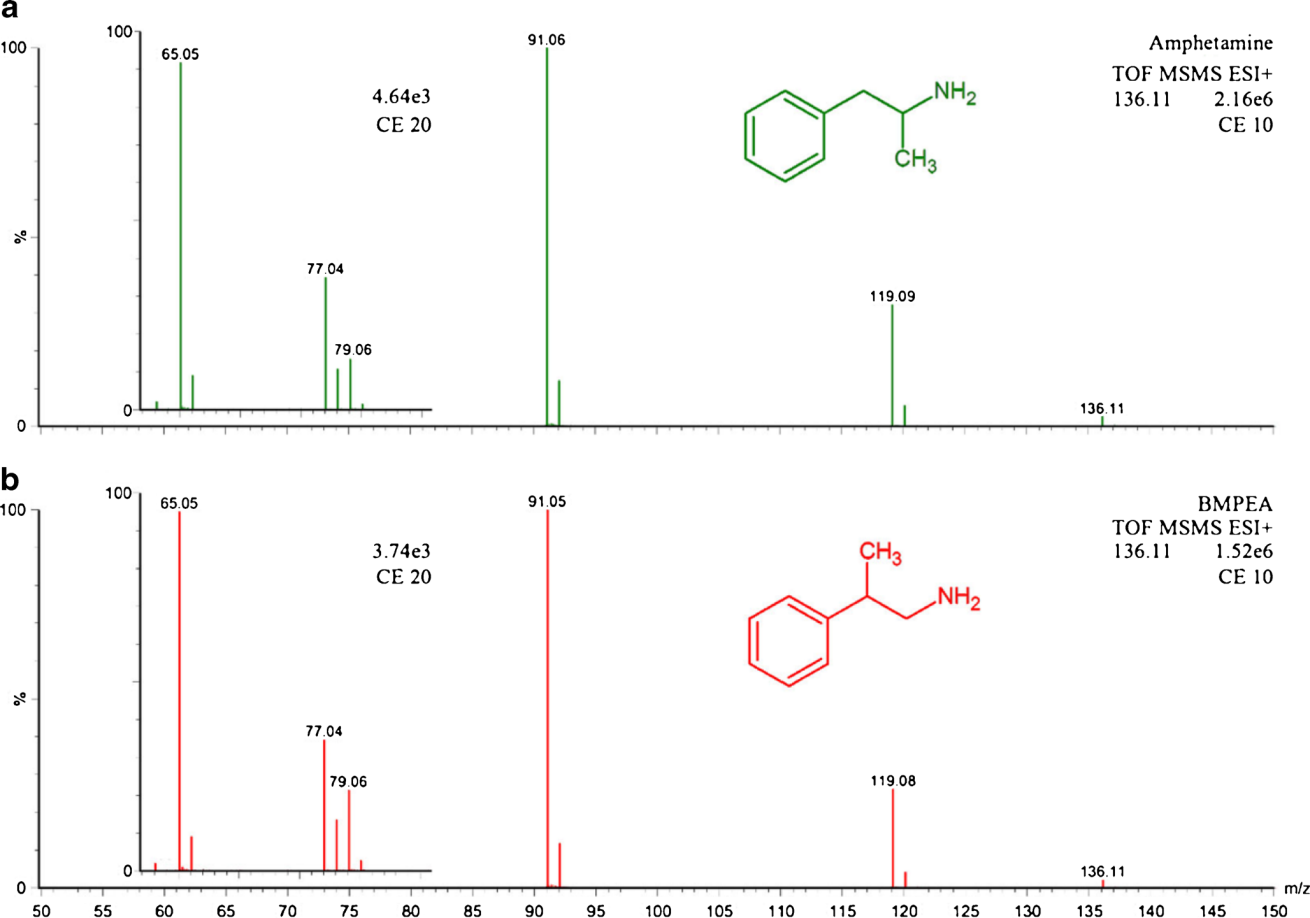
ToF MS/MS spectra for the *m*/*z* 136.11 precursor ions of amphetamine (**a**) and BMPEA (**b**)

**Fig. 2:**
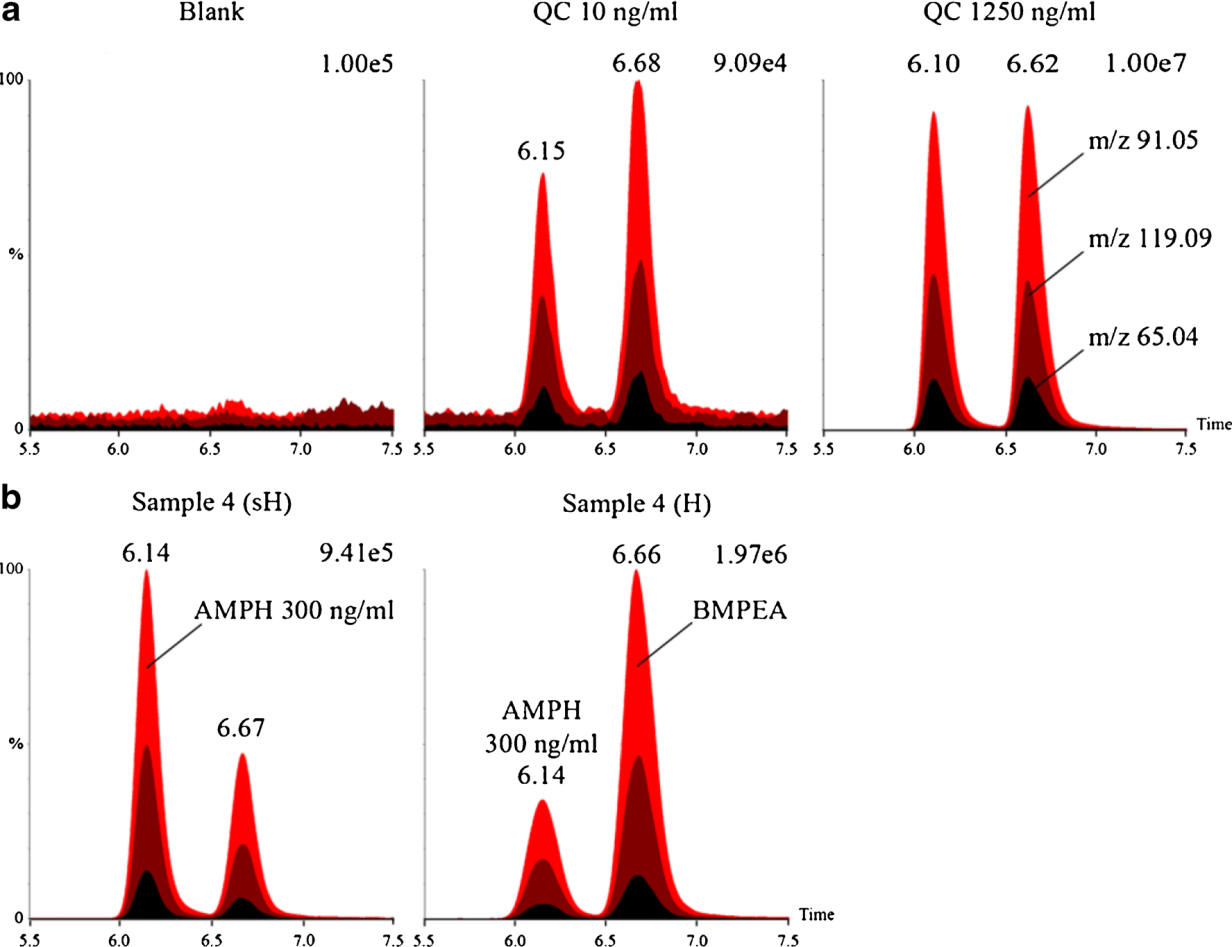
Discrimination capabilities of the method at different concentration levels (**a**) and chromatograms of a real case sample prepared with hydrolysis (*H*) or shortened hydrolysis (*sH*) of metabolic conjugates (**b**). Amphetamine (*AMPH*) was used as an internal standard to quantify BMPEA concentration in excretion urines. Extracted ion chromatograms were recorded for the precursor ion of *m*/*z* 136.11

Initial analysis of a received urine sample followed screening procedures for in-competition testing. An intense signal was observed in the chromatogram of amphetamine (MRM 136.11 > 91.05) from the UPLC/MS/MS screening for stimulants and narcotics. In this case, however, the athlete declared the use of the No-shotgun nutritional supplement which had BMPEA listed as an additive in the nutrition label. As amphetamine and BMPEA are positional isomers, it was important to evaluate their fragmentation patterns. The CID spectra were obtained by using the high-resolution time-of-flight technology (at least 14000 FWHM) and showed that both compounds fragmented into ions of the same *m*/*z* values (Fig. 1) which hampered their possible direct discrimination. Therefore, amphetamine and BMPEA needed to be resolved chromatographically for their unequivocal identification. Several different columns, mobile phases, and gradients were tested (not shown), and the best results were obtained with isocratic separation (0.1 % formic acid in methanol/0.1 % formic acid in water, *v*/*v*, 1/9) performed on an HSS T3 column (Fig. 2). Performance of the method was subsequently evaluated in compliance with the International Standard for Laboratories and EURACHEM guidelines [23, 24].

### Method validation

#### Selectivity

Selectivity of the method was assessed by the analysis of 10 blank urines. Evaluation of chromatograms recorded for three selected precursor ion-product ion transitions at the retention times of amphetamine and BMPEA (±1.0 min) showed the absence of any interfering components. It is also important to note that the sample preparation protocol of the confirmatory method was developed (with minor modifications) based on a highly selective screening procedure for amphetamine that had been used for analysis of at least a few thousands of samples. The sole drawback of the screening procedure in relation to the data presented here is the lack of BMPEA and amphetamine chromatographic separation. To provide additional information on the selectivity of the confirmatory method, 20 routine screening samples that had been deemed negative were additionally tested. Analysis of obtained chromatograms showed no interfering peaks even though the screening samples were approx. 33 times more concentrated than those prepared for confirmatory analysis. This data indicates that the developed method is highly selective for determining the presence of amphetamine and BMPEA in urine.

#### Discrimination capability and limit of detection

The capability of the method to discriminate between amphetamine and BMPEA at different concentrations was evaluated by the analysis of six blank urine samples spiked with these substances at four different levels: 10 ng/ml (0.1 MRPL), 50 ng/ml (0.5 MRPL), 250 ng/ml (2.5 MRPL), and 1,250 ng/ml (12.5 MRPL). This range was chosen based on our experience with amphetamine findings in routine anti-doping testing as well as in order to meet the WADA requirements concerning detection of stimulants in urine [20]. As shown in Fig. 2A, the method allowed for differentiation between amphetamine and BMPEA within the tested concentration range. LODs for both compounds were established at 10 ng/ml as it was the lowest concentration targeted in validation. Owing to the fact that anti-doping laboratories should not report the presence of stimulants in urine samples below 50 ng/ml [20], this was deemed satisfactory.

#### Recovery, matrix effect, and process efficiency

Recovery (RE), matrix effect (ME), and process efficiency (PE) were determined at two concentration levels in six different urine matrices on two different days. As shown in Table 1, RE, ME, and PE values were similar for amphetamine and BMPEA. Interestingly, the observed PE values close to 100 % are a result of antagonistic effects of low REs and signal enhancement (positive influence of matrix on the ionization process). The latter phenomenon is rather rarely observed in ESI-based analyses, as this technique is known to lead mostly to signal suppression [22]. As signal enhancement was found in all matrices tested, it is likely caused by residual compounds of endogenous origin. Importantly, variability of matrix effects (SD_ME_) established for the six individual matrices is close to 15 % (Table 1), the acceptable limit for this parameter [25]. Owing to the fact that RE, ME, and PE parameters for amphetamine and BMPEA are comparable, it is possible to use amphetamine as the internal standard for BMPEA quantitation in urine and vice versa.

**Table 1:** Recovery (RE), matrix effect (ME), and process efficiency (PE) parameters established for the method SD_ME_ is the standard deviation of the ME parameter

Concentration (ng/ml)	RE (%)	ME (%)	SD_ME_ (%)	PE (%)
BMPEA				
50	77.33	125.17	8.21	97.00
1,250	65.86	141.07	15.59	93.40
Amphetamine				
50	77.19	120.11	9.78	92.89
1,250	64.66	139.35	13.55	90.67

#### Carry-over

Carry-over was evaluated by injection of three blank urine samples directly after samples spiked post-extraction with amphetamine and BMPEA at 1,250 ng/ml. Visual inspection of chromatograms of the blank urines revealed no noticeable carry-over (<0.1 %).

### Application to real case samples

Application of the developed procedure resulted in the identification of BMPEA in the suspected sample (data not shown), and consequently, the first case of BMPEA doping was reported (2010). Additionally, the method of chromatographic separation allowed for the detection of BMPEA in the No-shotgun supplement, in which it was estimated to be present at approx. 770 μg/g (16 mg per serving; our unpublished results). Afterwards, all urine samples suspected for amphetamine/BMPEA were routinely analyzed by the described method revealing additional seven cases of doping with BMPEA.

To provide the first insights in the process of BMPEA excretion, four real case samples were used to investigate the influence of hydrolysis of phase II metabolic conjugates on method performance. Each excretion sample was prepared in duplicate for which one was hydrolyzed for a shorter time (acidic hydrolysis may have occurred in these samples only for 20 min at ambient temperature during the cleanup extraction, whereas the other samples were hydrolyzed for additional 30 min at 105 °C). Concentrations of BMPEA were estimated by using amphetamine (internal standard) spiked at 300 ng/ml (Fig. 2B). The analysis showed that shortening the duration of the hydrolysis step decreased the signal recorded for BMPEA up to 5-fold (Table 2). This data shows that BMPEA may be eliminated as direct conjugates, e.g., glucuronides, synthesis of which bypasses the phase I metabolic pathways. Moreover, these metabolites seem to be present in the urine sample with high *H*/sH ratio (approx. 5) at much higher levels than free BMPEA. On the other hand, longer hydrolysis caused only a slight increase in the BMPEA concentrations measured in the other samples (*H*/sH value close to 1; Table 2). This may indicate a negative influence of the matrix on deconjugation efficiency and/or that unchanged BMPEA may be more abundant in these samples than its phase II conjugates. The latter possibility was tested by a dilute-and-shoot approach.

**Table 2:** Estimated concentrations of BMPEA in excretion samples

Sample		Estimated concentration (ng/ml)					
	*H*/sH ratio	*H*	CV (%)	sH	CV (%)	Dilute and shoot	CV (%)
1	1.44	1,208	4.2	836	7.8	778	4.4
2	1.53	420	1.2	274	3.8	321	14.6
3	1.25	1,249	6.7	997	2.0	1,011	–^a^
4	5.61	881	3.7	157	4.5	158	6.5

*H* hydrolysis, *sH* shortened hydrolysis

^a^Mean of two measurements

### Detection of free BMPEA in excretion samples by dilute-and-shoot method

**Fig. 3:**
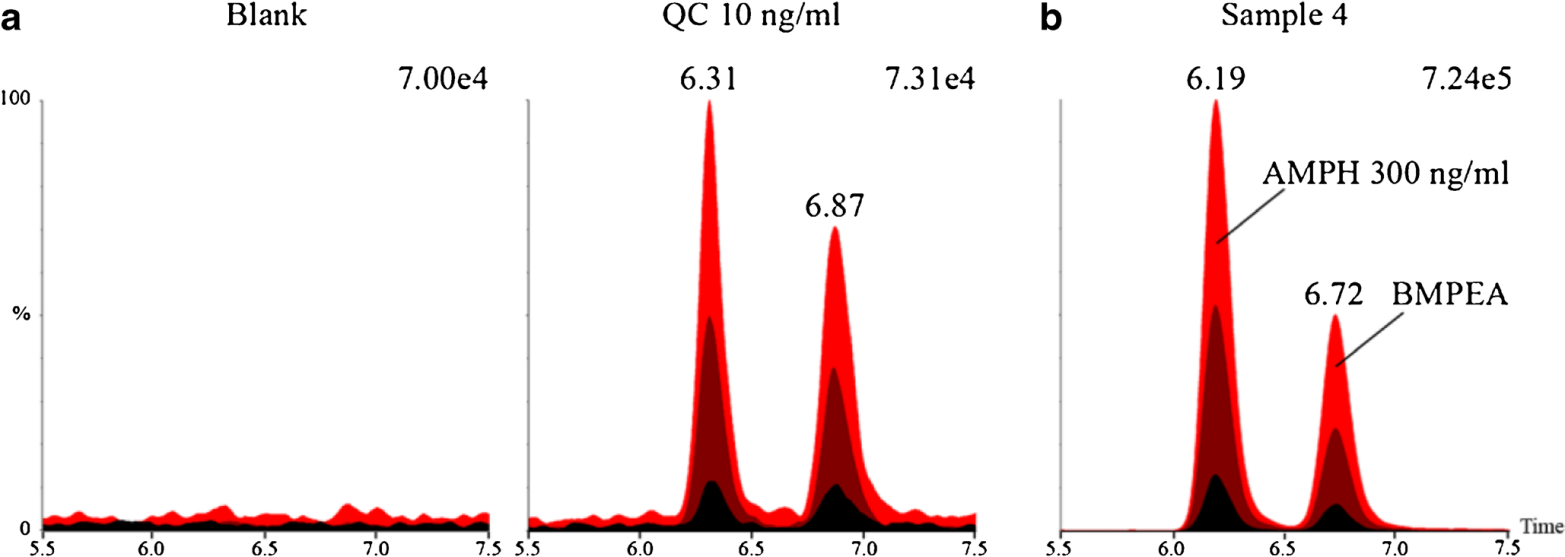
Analytical performance of the dilute-and-shoot method (**a**) and a chromatogram of excretion sample (**b**). Amphetamine (*AMPH*) was used as an internal standard to quantify BMPEA concentration and to correct for matrix-dependent shifts of BMPEA retention time

To test whether BMPEA may be eliminated in a free form, excretion samples were analyzed by using a dilute-and-shoot method. This procedure was demonstrated to be selective by analysis of 20 different urine samples which revealed no interfering peaks at the retention times of amphetamine and BMPEA (±1.0 min; Fig. 3A). Limits of detection for both substances were established at 10 ng/ml as this was the lowest concentration tested (Fig. 3A). The stability of retention times was, however, rather poor, indicating a strong influence of the matrix on chromatographic separation (Fig. 3A, B). Thus, amphetamine was used as the internal standard to estimate BMPEA concentration and to correct for instability of BMPEA retention time. The analysis showed that unchanged BMPEA was present in all excretion samples in concentrations similar to those estimated in the corresponding samples prepared with shortened hydrolysis (Table 2, Fig. 3B). Thus, our data indicates that BMPEA may be eliminated unchanged and as conjugates, and that the elimination profile of BMPEA may undergo significant changes, perhaps similar to those already described for amphetamine [26, 27]. Additionally, the high structure similarity between amphetamine and BMPEA suggests that both compounds may also share some of their phase I metabolic pathways, e.g., aromatic hydroxylation [26, 27]. This hypothesis, however, requires further research as such putative BMPEA metabolites were not targeted in this study.

## Conclusions

The analytical procedure for discrimination of amphetamine and BMPEA in urine samples presented here was proven to be simple and rapid and was validated in compliance with appropriate guidelines and WADA requirements. The limits of detection were established at 10 ng/ml, and discrimination capabilities were demonstrated to be satisfactory up to the concentration of 1.25 μg/ml. The analytical performance related to matrix effects, recovery, and carry-over was acceptable and very similar for both compounds, indicating that one substance may be used as the internal standard for quantitation of the other. The fit for purpose of the method was also demonstrated by analysis of real case samples and resulted in reporting of eight BMPEA doping cases. Further investigation of excretion samples provided first insights into BMPEA metabolism and showed that it may be eliminated unchanged and in the form of its phase II conjugates (phase I metabolites were not targeted in this study). Interestingly, the relative concentrations of both fractions may considerably differ in a given sample. Consequently, omission of the hydrolysis step may produce false-negative results, as it was shown to increase BMPEA concentration even up to 5-fold. The fact that both isomers fragment yielding comparable CID spectra indicates that insufficient chromatographic separation may result in misidentification. Thus, testing laboratories should interpret their LC/MS/MS-based amphetamine and BMPEA findings with great care. This is of utmost importance in the forensic field as BMPEA is not controlled by the public law, and its manufacturing, distribution, and use are legal.
